# Unraveling the drivers of forage quality variation in the Serengeti

**DOI:** 10.1002/ecy.70168

**Published:** 2025-07-23

**Authors:** Yuhong Li, Sanne Piek, Emilian P. Mayemba, Kelvin R. Shoo, Michiel P. Veldhuis, Han Olff

**Affiliations:** ^1^ Conservation Ecology Group, Groningen Institute for Evolutionary Life Sciences (GELIFES) University of Groningen Groningen The Netherlands; ^2^ Institute of Environmental Sciences University of Leiden Leiden The Netherlands

**Keywords:** herbivore coexistence, intraspecific trait variation, nutrient stoichiometry, plant nutrient content, resource partitioning, savanna, spatial heterogeneity, species turnover

## Abstract

Variation in forage quality is a key dimension of herbivore resource partitioning, but the main determinants of such variation across environmental gradients remain poorly understood. It is especially unclear how much variation in plant nutrient contents and stoichiometry is driven by plant species turnover versus by intraspecific variation across sites. We investigated variation in forage quality across nine sites along a key environmental gradient of increasing rainfall and decreasing soil fertility in the Serengeti National Park, Tanzania. We compared leaf elemental contents of nitrogen (N), phosphorus (P), potassium (K), calcium (Ca), magnesium (Mg), and sodium (Na) and three nutrient ratios (N:P, Ca:P, and K:Na) between sites, between species within the same site, and between sites within the same species. Site‐average N, P, and K leaf contents decreased with increasing rainfall and decreasing soil fertility. The decline in N and K was primarily associated with species turnover, with their contents remaining relatively stable within species. The decline in P was associated with a combination of species turnover and intraspecific variation, with intraspecific P content decreasing strongly with increasing rainfall (decreasing soil fertility) across sites. Variation in site‐average Ca, Mg, and Na leaf contents did not significantly correlate with rainfall or soil fertility and was mainly explained by species turnover between sites. Comparing leaf nutrient content and ratios to literature‐derived nutritional requirements for large herbivores suggests that Na is severely limiting in this ecosystem. K seems sufficient everywhere, and the other elements are moderately limiting. If Serengeti herbivores rely on plants for their nutrient intake and are nutrient‐limited, these results suggest herbivores with high N, Ca, or Mg requirements should optimize their diet by selecting particular species, relatively independent of sites. Herbivores with a high P requirement can instead best select particular sites, relatively independent of plant species. To obtain sufficient Na, herbivores can target particular species at particular sites. Thus, resource partitioning among Serengeti herbivores may occur at different levels for different elements. Interspecific variation in herbivore nutrient requirements would then drive resource partitioning both across sites (for P and Na) and between plant species (N, Ca, Mg, and Na).

## INTRODUCTION

Resource partitioning can be a key contributor to biodiversity in many ecosystems, as it allows multiple species within a guild to coexist by utilizing similar resources differently (Pansu et al., 2022; Pringle et al., 2019; Root, 1967). Among herbivores, food partitioning based on resource quality has been recognized as an important mechanism supporting species coexistence (Bell, 1971; Daskin et al., 2023; Du Toit & Olff, 2014; Geist, 1974; Potter et al., 2022). Because more nutritious resources are scarcer across landscapes and therefore require greater foraging effort, herbivores, guided by optimal foraging principles, tend to avoid pursuing more nutrient‐rich food and instead target resources that meet their specific nutrient needs (Belovsky, 1997; Ritchie & Olff, 2004). Therefore, interspecific variation in herbivores' nutritional requirements can lead different species to exploit food resources of varying quality and quantity, reducing competition and fostering coexistence (Prins & Olff, 1998; Ritchie & Olff, 2004). Such resource partitioning has been attributed to spatial variations in forage quality and quantity (Olff et al., 2002; Ritchie & Olff, 2004). Changes in forage nutrient content across environmental gradients have been documented in multiple savanna ecosystems (Hopcraft, 2010; Hopcraft et al., 2012; Skidmore et al., 2010; Veldhuis, Hulshof, et al., 2016). Nevertheless, the drivers of these variations in forage quality remain insufficiently understood.

In principle, changes in plant nutritional traits between plant communities could arise either from species turnover (i.e., changes in dominance of species with different intrinsic nutrient content) or from intraspecific variation (i.e., same species composition but varying nutrient content within species). However, the relative contribution of these two factors to nutrient landscape variation remains largely unquantified. Forage nutrient (elemental) content of a plant community is commonly measured using pooled samples of mixed plant species (Anderson et al., 2007, 2010; Hopcraft et al., 2012; McNaughton, 1988; Veldhuis, Fakkert, et al., 2016), which does not infer this mechanism underlying nutrient variation across communities. In particular, it is often assumed that nutrient content remains constant within a species across sites. For example, species composition is commonly used as an indicator of forage quality (Anderson et al., 2007; Sianga & Fynn, 2017), and fixed nutrient values are assigned to plant taxa in herbivore diet quality assessment (Lan et al., 2021; Pansu et al., 2019; Potter et al., 2022). However, trait variation within species can be as large as the variation between species (Siefert et al., 2015; Umaña & Swenson, 2019). Therefore, to better understand the mechanism underlying the variation in forage quality across landscapes (between sites), it is crucial to quantify the contributions of both species turnover and intraspecific variation.

**FIGURE 1:**
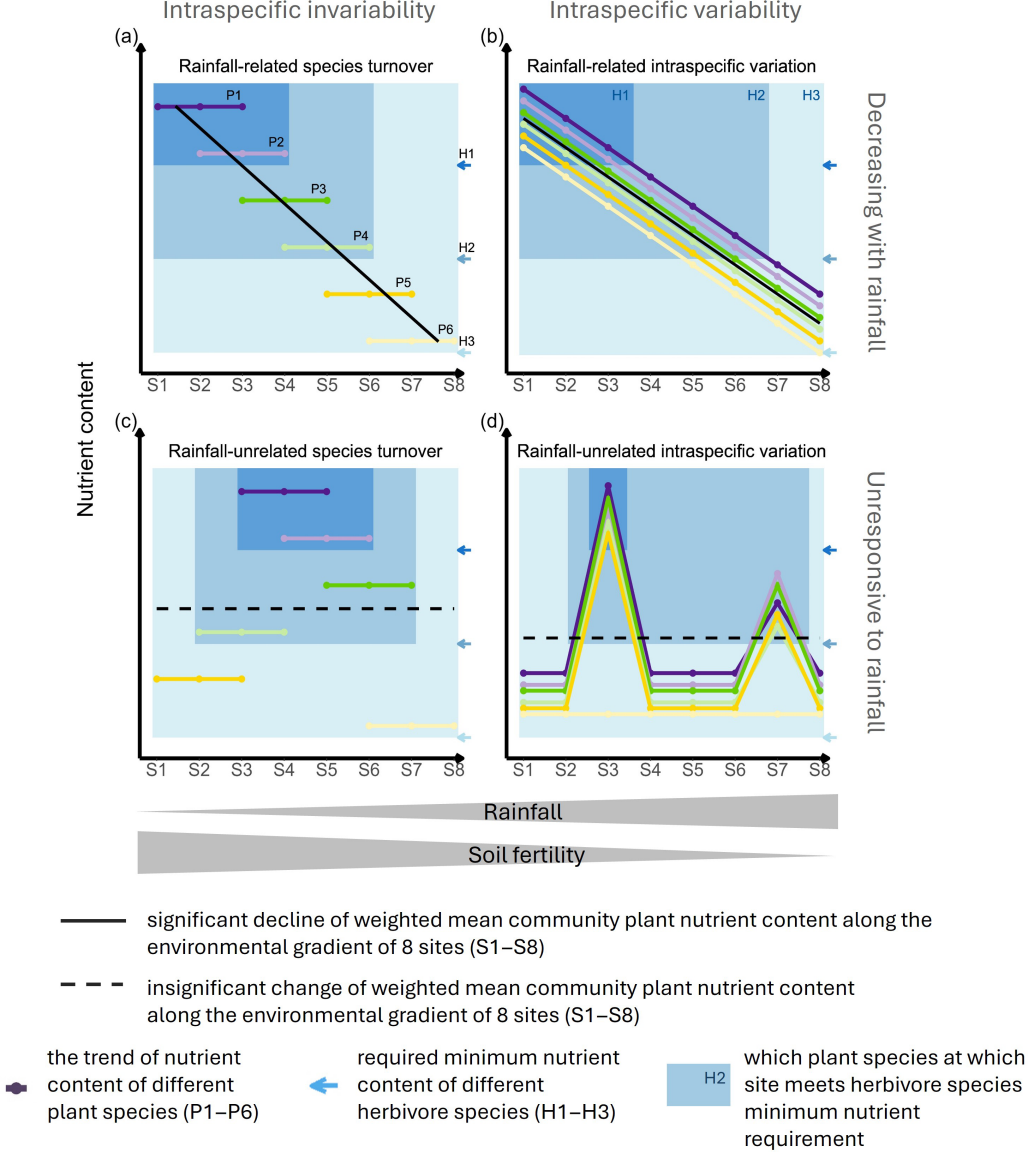
Four hypotheses explaining variation in plant nutrient content across species and sites. This conceptual figure illustrates three herbivores that vary from high nutrient requirements (H1) to intermediate (H2) to low nutrient requirements (H3), with the boxes showing which of six plant species P1 (highest quality) to P6 (lowest quality) are selected on eight different sites from S1 to S8. The four hypotheses propose alternative explanations for how forage nutrient content varies along a rainfall‐soil fertility gradient, based on the level of within and between species variation in nutrient content and the effect of rainfall and soil fertility: (a) overall vegetation nutrient content decreases with increasing rainfall (represented by the solid black trendline), which is driven by species turnover along the rainfall gradient from high‐quality species to low‐quality species while nutrient content remains constant within species; (b) overall vegetation nutrient content declines with increasing rainfall (the solid black trendline), explained by intraspecific variation of nutrient content while species composition remains the same; (c) overall vegetation nutrient content is unrelated to rainfall (the dashed black trendline), because turnover of species with fixed nutrient contents is unrelated to rainfall; (d) overall vegetation nutrient content is unrelated to rainfall (the dashed black trendline), because high contents are found for specific species at specific sites independent from rainfall and without much change in species composition. The main consequences of these four hypotheses for herbivore food selection are: in hypothesis (a), selective herbivores choose particular plant species at high quality sites, and these high quality sites are predictably found at low rainfall, high soil fertility; in hypothesis (b), selective herbivores choose high‐quality sites (and can eat any plant species there), and these high‐quality sites are predictably found at low rainfall, high soil fertility; in hypothesis (c), selective herbivores choose particular plant species at high‐quality sites, but such high‐quality sites are not predictable from the rainfall‐soil fertility gradient; in hypothesis (d), selective herbivores choose high‐quality sites (and can eat all plant species there), but such high‐quality sites are not predictable from the rainfall‐soil fertility gradient.

Rainfall and soil fertility are crucial determinants of forage quality in arid and semi‐arid African biomes and are often negatively correlated given the same soil type (Breman et al., 1979; Jager, 1982). This results in nutrient‐rich but limited forage in areas with low rainfall and high soil fertility, and nutrient‐poor but plentiful forage in areas with high rainfall and low soil fertility (Breman et al., 1979; Olff et al., 2002). If this forage nutrient variation is solely driven by plant species turnover, we expect to find a transition in species composition from inherently nutrient‐rich species at low rainfall to nutrient‐poor species at high rainfall, with intraspecific nutrient content remaining relatively constant (Figure 1a). If intraspecific variation is the main driver, we expect that the nutrient content of each species decreases with increasing rainfall, with a lesser effect of species turnover (Figure 1b). In reality, a combination of these scenarios is likely, where some degree of species turnover is mixed with some degree of intraspecific nutrient variation (Lepš et al., 2011). In addition, forage nutrient content may not be directly related to the rainfall–soil fertility gradient. For example, the impact of local biological interactions could override the regional‐scale effect of abiotic factors (Cromsigt et al., 2017; Veldhuis, Hulshof, et al., 2016). In this scenario, the variation in forage quality across the landscape can be the result of changes in species composition (Figure 1c) or intraspecific variation (Figure 1d). Altogether, this leads to four hypotheses on variation in forage nutrient content based on its correlation with the rainfall–soil fertility gradient and the extent of intraspecific variability, all of which will be tested in this study (see caption in Figure 1).

Nutrient requirements of herbivores vary among species (Demment & Van Soest, 1985; Geist, 1974; Illius & Gordon, 1992). For example, nitrogen content in diets is negatively related to species body mass (Daskin et al., 2023; Potter et al., 2022), while sodium intake per unit body mass is positively related to body mass (Duvall et al., 2023). Combining variation in herbivore nutrient requirements with forage nutrient content across plant species and sites leads to different scenarios on how herbivores select and partition food across landscapes (see blue arrows and boxes in Figure 1). Based on the hypothesis of rainfall‐related species turnover (Figure 1a), a herbivore with high nutrient requirements (H1) would be highly selective and consume only plant species 1 and 2 (P1 and P2); a herbivore with intermediate nutrient needs (H2) would select plant species 1, 2, 3, and 4 (P1–P4), while a herbivore with low nutrient requirements (H3) would be a generalist and have the flexibility to consume all plant species (P1–P6). In this scenario, herbivores select food by plant species, and this taxon‐based food partitioning is organized along the rainfall–soil fertility gradient. Alternatively, under the hypothesis of rainfall‐related intraspecific variation (Figure 1b), all three herbivore species can consume all six plant species, but they must go to specific sites to access forage that meets their nutritional demands. Accordingly, herbivores select food by sites along the rainfall–soil fertility gradient. Similarly, in the hypothesis of rainfall‐unrelated species turnover (Figure 1c), herbivore species with different nutritional requirements select food by species of which nutrient content is unrelated to rainfall and soil fertility. In the hypothesis of rainfall‐unrelated intraspecific variation (Figure 1d), herbivores select food by sites, which are unrelated to rainfall and soil fertility. Therefore, how herbivore species select and partition food resources depends on how plant nutrient content responds to the environmental gradients, both within and across plant species.

If herbivores are resource‐limited, site and species selection by herbivores is expected to optimize their resource intake. Theory suggests such selection should be strongest for those resources that are most limiting (Tilman, 1982). This is particularly important for herbivore food selection because herbivores have a significantly higher nutrient content (nutrient: carbon ratios) in their body chemical composition compared to their plant food (Sterner & Elser, 2002; Van Soest, 1994). When nutrients are limiting, this disparity drives herbivores to seek out forage with high nutrient content (Grant & Scholes, 2006; McNaughton, 1988). However, research on plant nutrient content and its variation across the landscape has mainly focused on the elements critical for plant growth, such as nitrogen, phosphorus, and potassium, whereas elements that are less crucial for plant growth but vital for animal nutrition have been less systematically studied, such as calcium, magnesium, and sodium (Hopcraft et al., 2012; Koerselman & Meuleman, 1996; Treydte et al., 2013; Veldhuis, Fakkert, et al., 2016). Consequently, our understanding of how well forage meets herbivore nutrient requirements—specifically, which of these six essential nutrients is most limiting—is limited (Simpson et al., 2004). To identify the key elements limiting herbivores and impacting their food selection, it is essential to quantify the variation in forage element contents across landscapes and link it to estimates of herbivore nutrient requirements.

Besides the quantity of specific nutrients, the relative proportions of different nutrients—ecological stoichiometry—play an important role in animal nutrition (Kaspari, 2020; Sitters & Olde Venterink, 2021; Sterner & Elser, 2002). For instance, the ratio of calcium to phosphorus intake should be maintained around 2:1 for ruminants because an excess of phosphorus would hinder the absorption of calcium by the gut (Manston, 1967). Similarly, sustaining a balanced intake of sodium relative to potassium is necessary to support the osmoregulatory function of sodium‐potassium pumps in animal cells, and excessive intake of potassium can trigger a cascade of fatal ionic imbalances (Kaspari, 2020). Furthermore, the intake ratio of nitrogen to phosphorus is predicted to decrease with body mass because the demand for phosphorus required in skeletal development outpaces the need for nitrogen required in protein synthesis as body mass increases (Elser et al., 1996; Le Roux et al., 2020; Sitters et al., 2017). Comparing ratios of nutrients present in plants to herbivore intake requirements enables us to identify which nutrients are likely to be more limiting and therefore more important for diet selection.

To improve our understanding of how large herbivores might partition resources at the element, plant species, and site levels, we investigated the drivers of variation in forage nutrient content across a savanna landscape in the Serengeti National Park, a protected area characterized by a high diversity of coexisting mammalian herbivores (Mduma & Hopcraft, 2008). We measured the contents of six essential elements—nitrogen (N), phosphorus (P), potassium (K), calcium (Ca), magnesium (Mg), and sodium (Na)—in leaves of a wide range of herbaceous species at nine sites across a rainfall‐soil fertility gradient (annual rainfall range: 681–999 mm). For each element, we examined how site‐average element content varies along the rainfall‐soil fertility gradient and quantified the relative contributions of species turnover effect and intraspecific variation to the total element content variation across sites. With this information, we discriminated among the four hypotheses outlined in Figure 1, separately for each element. Last, we compared site‐ and plant species‐specific element content and N:P, Ca:P, and K:Na ratios against literature‐derived nutritional requirements of herbivores of different body masses to estimate to what extent nutrient content meets herbivore nutritional needs.

## METHODS

### Study sites

**FIGURE 2:**
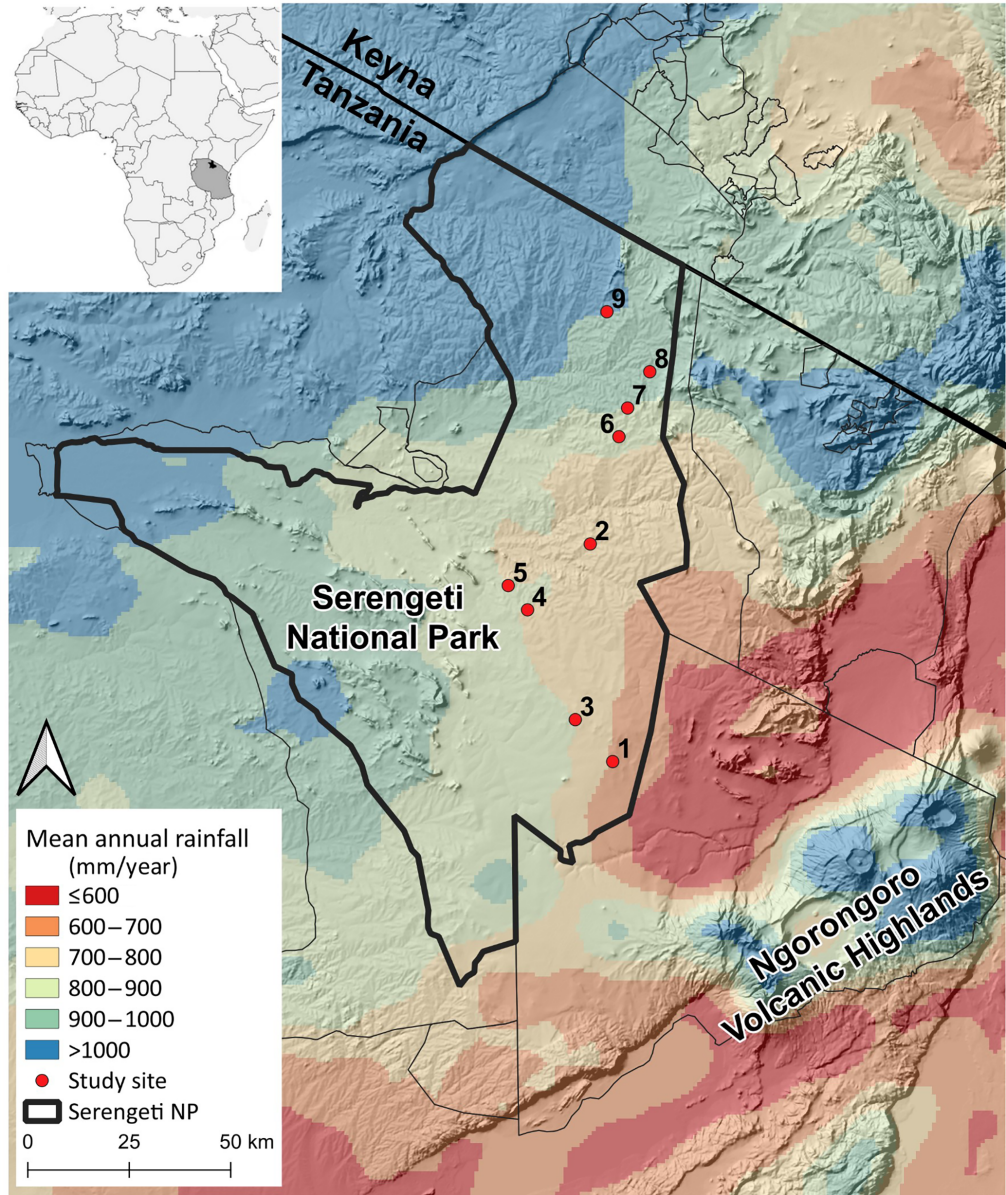
Spatial distribution of the nine study sites across the rainfall‐soil fertility gradient in the Serengeti National Park, Tanzania. The sites were numbered sequentially based on their rainfall levels, from lowest to highest, as calculated using the CHIRPS dataset (Climate Hazards Group InfraRed Precipitation with Station data) averaged over the period from 2000 to 2021.

This study was conducted in the Serengeti National Park (SNP; 2.33° S, 34.83° E), a core protected area spanning 12,763 km^2^ within the Greater Serengeti Ecosystem in northern Tanzania. The Serengeti Ecosystem hosts a diverse array of 29 large mammalian herbivore species (tab. A.2 in Mduma & Hopcraft, 2008). The climate and geology within the SNP are impacted by the Ngorongoro Volcanic Highlands, situated southeast of the park (Figure 2). These highlands induce a strong rain shadow effect, resulting in a steep gradient in annual rainfall across the SNP, ranging from less than 500 mm in the southeast to over 1000 mm in the north‐west (Norton‐Griffiths et al., 1975). Rainfall predominantly occurs during the short wet season (November–December) and the long wet season (March–May), with a pronounced dry season (June–October) (Norton‐Griffiths et al., 1975). Intermittent deposition of mineral‐rich volcanic ash from the Ngorongoro highland volcanoes overlays the old, infertile soil across the SNP (De Wit, 1978; Jager, 1982). The influence of volcanic ash diminishes towards the north‐west due to increased distance from the volcanic source (Jager, 1982). In southeastern SNP, the accumulation of volcanic ash rich in calcium carbonate leads to the formation of a dense, compacted soil layer (i.e., hardpan) that is impervious to water and root penetration, which results in a landscape of treeless grasslands in that area (De Wit, 1978). This geological attribute results in a strong inverse correlation between soil nutrient availability and rainfall (Jager, 1982).

The compound gradient of rainfall and soil fertility results in landscape‐scale spatiotemporal heterogeneity and dynamics in forage quality and availability, driving the annual migration of millions of wildebeest and zebras southwards for breeding during the wet season and northwards for abundant forage during the dry season (Holdo et al., 2009). Vegetation composition varies across the region, with grasslands dominating the south and *Acacia* and *Commiphora* savanna prevailing in the central and northern areas (Herlocker, 1976). Additionally, with increasing grass productivity, fire return intervals shorten from more than 20 years in the plains to every 2 years in the northern savannas (Veldhuis et al., 2019).

To assess how forage nutrient content varies along the rainfall–soil fertility gradient, we used rainfall as a proxy for this environmental variation. We generated a mean annual rainfall map utilizing the CHIRPS dataset (Climate Hazards Group InfraRed Precipitation with Station data) spanning from 2000 to 2021 (Funk et al., 2015). Nine sites were selected along the rainfall–soil fertility gradient, ranging from 681 to 999 mm year^−1^ on average over this period (Figure 2). All sites are situated on granite or gneiss parent materials overlaid with a layer of volcanic ash that decreases in thickness from south to north (Appendix S1: Figure S1). Moreover, all sites were positioned on mid‐slope catena elevations to standardize the influence of topography on vegetation and soil properties (Hamilton et al., 2001; Jager, 1982; McNaughton, 1985).

### Plant leaf sampling

We focused on herbaceous plants (i.e., grasses and forbs) for two main reasons. First, the Serengeti large herbivore community is dominated by species that primarily consume grasses and forbs (Mduma & Hopcraft, 2008), suggesting that resource partitioning among herbaceous plants is more general than among woody plants. Second, the sampling method designed for herbaceous plants—small sampling plots to keep herbaceous species identification and quantification manageable—is far less suitable for woody vegetation due to low encounter rates of woody species, which are scattered across the continuous herbaceous layer. To understand the distribution of herbaceous species and their nutrient content along the rainfall‐soil fertility gradient, we conducted a vegetation survey along a single 100‐m transect at each of the nine sites in the 2022 wet season (March and April). At intervals of 5 m along the transect, we established sampling plots of 35 × 35 cm^2^, resulting in 21 plots per transect. Within each sampling plot, we determined which plant species were present. Nomenclature followed the World Checklist of Vascular Plants (WCVP) database (https://powo.science.kew.org/, accessed in March 2022). Given the wide variation in height observed among herbaceous plants in the field, ranging from 3 to 170 cm, we recorded the height layers occupied by each plant species at every sampling plot, categorizing them into four predefined levels (0–10 cm, 10–30 cm, 30–50 cm, 50–200 cm). Subsequently, we quantified the layer abundance of each species at a site as the total number of layers it occupied across all sampling plots along the transect (at most 21 plots × 4 layers). We used this 100‐m transect approach to characterize the average community composition of sites due to the relatively high heterogeneity within sites, for example, as determined by tree canopies, animal disturbances (such as burrows), herbivore paths, and termite mounds.

To determine plant nutrient contents, we collected leaf samples for the most abundant plant species. The sample of each plant species at a site consisted of pooled fully green leaves from multiple mature individuals (≥15) collected across the site (the 100‐m transect) so that it would not be biased by individual growing microhabitats. Leaf samples were oven‐dried at 70°C to a constant mass, ground, and homogenized using a Foss Cyclotec grinder (Hillerød, Denmark) for further chemical analyses. In total, 75 leaf samples were collected, representing together 45 different grass and forb species and an average of 56% of the total leaf layer abundance at each site. Among these species, 10 plant species were collected at three or more sites, enabling us to compare nutrient content within species across sites.

### Elemental content measurement

For each leaf sample, we measured the elemental contents (in percentage) of N, P, K, Ca, Mg, and Na. Total N content was estimated with a near‐infrared (NIR) spectrophotometer (Bruker Optik GmbH, Ettlingen, Germany), using a multivariate calibration made by 107 mixed‐species herbaceous plant samples that were collected earlier in the Serengeti ecosystem and measured both on the NIR and CHNS EA1110 elemental analyzer (Carlo‐Erba Instruments, Milan, Italy). Cross‐validation indicated sufficient accuracy in NIR‐predicted N content (*R* ^2^ = 0.92, *n* = 107). To measure the other element contents, 100‐mg subsamples were digested in a 2‐mL mixture of 4:1 HNO_3_/HCl in a closed Teflon cylinder at 140°C for 7 h and subsequently diluted with 8‐mL demineralized water. Total P content was determined using a spectrophotometer colorimetric approach (UV‐1601PC; Shimadzu Corp., Kyoto, Japan) with the ammonium molybdate method (Murphy & Riley, 1962). Total K, Ca, Mg, and Na contents were measured using an atomic absorption spectrophotometer (1100B Spectrometer, PerkinElmer Inc., Waltham, MA, USA) after 1% LaNO_3_ was added to the solution to break down the phosphate bonds.

### Herbivore nutrient requirements

To get an indication of whether and which leaf elemental contents are potentially limiting for herbivores—and thus important for dietary selection—we collected data from the literature on nutrient requirements of herbivores. This was done for species of different body sizes because body mass has been shown to correlate with nutrient requirements of multiple elements (Duvall et al., 2023; Potter et al., 2022). Due to the limited information available on the nutrient requirements of wild herbivores (except for wildebeest), we used the nutritional intake needs of sheep *Ovis aries* to represent smaller herbivores (e.g., Grant's gazelle *Gazella granti* and impala *Aepyceros melampus*), wildebeest *Connochaetes taurinus* for intermediate‐bodied herbivores (e.g., topi *Damaliscus korrigum* and hartebeest *Alcelaphus buselaphus*), and cattle *Bos taurus* for large‐bodied herbivores (e.g., buffalo *Syncerus caffer*). We estimated these nutrient requirements during both lactating and non‐lactating stages because needs during these two stages vary significantly. For the nutrient ratios, we only used the estimated Ca:P intake ratio required by lactating wildebeest and the N:P and K:Na intake ratios necessary for lactating cattle because we could not find specific ratios for each herbivore species at the non‐lactating stage. We compared these estimated herbivore nutrient needs (Table 1) to leaf nutrient contents and nutrient ratios across sites and plant species.

**TABLE 1:** Literature‐derived nutritional requirements of different‐sized herbivores.

Common name (body mass) and life stage	N %	P %	K %	Ca %	Mg %	Na %	N:P	Ca:P	K:Na
Cattle (600 kg)									
Lactating	1.71^a^	0.21^a^	0.69^b^	0.51^b^	0.21^b^	0.12^b^	6–8^a^	…	1–5^c^
Non‐lactating	1.40^a^	0.16^a^	0.58^b^	0.27^b^	0.18^b^	0.09^b^	…	…	…
Wildebeest (143 kg)									
Lactating	…	0.39^d^	…	0.34^d^	…	0.05^d^	…	1–2^e^	…
Non‐lactating	…	0.19^d^	…	0.12^d^	…	0.04^d^	…	…	…
Sheep (75 kg)									
Lactating	2.24^f^	0.32^b^	0.30^b^	0.28^b^	0.14^b^	0.08^b^	…	…	…
Non‐lactating	1.78^f^	0.11^b^	0.30^b^	0.14^b^	0.11^b^	0.07^b^	…	…	…

*Note*: The minimum nutrient contents in forage required for herbivore daily maintenance are presented for each of the six elements during the lactating and non‐lactating stages. Optimal nutrient ratios are presented as a range based on the nutritional requirements of lactating wildebeest (for Ca:P) and lactating cattle (for N:P and K:Na).

^a^ National Research Council (U.S.) (1996).

^b^ Suttle (2010).

^c^ Hu and Kung (2009) and Wildman et al. (2007).

^d^ Murray (1995).

^e^ Kreulen (1975).

^f^ Cannas et al. (2004) and Kyriazakis and Oldham (1993).

### Data analysis

#### Quantifying variation in nutrient content across sites and within species

To analyze the variation in forage nutrient content across the rainfall–soil fertility gradient, we calculated the specific weighted mean content of each element at a given site using the equation:

(1) $$\begin{array}{l} \text{Specific weighted mean}=\sum_{i=1}^{n}({\text{relative abundance}}_{i}\\ \;\times {\text{element content}}_{i-\text{site}}), \end{array}$$

where *n* represents the number of plant species collected at a site, the relative abundance of the *i*th species (${\text{relative abundance}}_{i}$) was calculated as the number of layers it occupied divided by the total number of layers of all plant species collected at that site, and the element content of the *i*th species (${\text{element content}}_{i-\text{site}}$) was site‐specific. For each element, we examined the variation of its specific weighted mean content along the rainfall‐soil fertility gradient using general linear models. To address the presence of extremely large observations in Ca, Mg, and Na contents—where these elements can be several times higher in forb species compared to grass species (Broadley et al., 2004)—we applied a log_10_ transformation to their values in the linear models to meet assumptions of normality.

To assess how leaf nutrient content varied within species across the rainfall–soil fertility gradient, we used the 10 representative species that were collected at three or more sites (see Appendix S1: Table S1 for the species names). We applied linear mixed‐effects models to analyze their elemental contents, with rainfall as a fixed factor and species as a random factor for the intercepts, using restricted maximum likelihood (REML) in the R package lme4 (Bates et al., 2023). For Ca, Mg, and Na, we normalized their contents by log_10_ transformation in the models.

In examining variation in nutrient stoichiometry, the site nutrient ratio was calculated by the site‐specific weighted mean of one element divided by that of the other element. Its relationship with rainfall was assessed using a general linear model. Variation in a nutrient ratio within species across the rainfall gradient was quantified using linear mixed‐effects models with species as a random factor for the intercepts in R package lme4 (Bates et al., 2023).

#### Decomposing variation into species turnover effect and intraspecific variation

To decompose the total variation of nutrient contents across sites into intraspecific variation and species turnover effect, we applied the method developed by Lepš et al. (2011). We first calculated the fixed weighted mean content of each element at each site using Equation (2).

(2) $$\begin{array}{l} \text{Fixed weighted mean}=\sum_{i=1}^{n}({\text{relative abundance}}_{i}\\ \;\times {\text{element content}}_{i-\text{fixed}}) \end{array}$$

All the components in Equation (2) remain the same as in Equation (1), except that the site‐specific nutrient content of the *i*th species, denoted as ${\text{element content}}_{i-\text{site}}$, is replaced with ${\text{element content}}_{i-\text{fixed}}$. The latter represents the mean nutrient content of the *i*th species, calculated by averaging the nutrient content of samples collected across sites. This value remains constant for each species and does not vary by site. As ${\text{element content}}_{i-\text{fixed}}$ is fixed for a species during the calculation of the fixed weighted mean, the sole source of the variation in fixed weighted mean across sites is changes in ${\text{relative abundance}}_{i}$, that is, species relative abundance (${\text{relative abundance}}_{i}$ equals to zero if *i*th species is absent). Correspondingly, species turnover effect can be quantified by the changes in fixed weighted mean across sites. Similarly, the differences between fixed weighted mean and specific weighted mean of a site can be used to quantify intraspecific variation because ${\text{relative abundance}}_{i}$ (i.e., species composition) for a site remains fixed, and the only variation source here is the difference between ${\text{element content}}_{i-\text{site}}$ and ${\text{element content}}_{i-\text{fixed}}$ which is variation within species. Then changes in specific weighted mean across sites present the total across‐site variation that encompasses both species turnover effect and intraspecific variation, along with their covariation. When the covariation is positive, it means that species turnover effect and intraspecific variation are positively correlated. For instance, at the site where element‐rich species dominate, individuals of the present species tend to be more element‐rich than the species' average level. Conversely, when the covariation is negative, it indicates that these two variation components are negatively correlated. For example, at the site where element‐rich species dominate, individuals of the present species tend to be more element‐poor than the species' average level. To assess the relative importance of each variation source, we then calculated the proportion of variation caused by species turnover and by intraspecific variation in the total across‐site variation.

Furthermore, we correlated the variations of the two sources with the rainfall‐soil fertility gradient using general linear models to investigate the extent to which species turnover effects and the intraspecific variation were related to rainfall (Lepš et al., 2011). Then, we compared the proportion of variation caused by species turnover and that caused by intraspecific variation to identify the primary cause of forage nutrient variation across communities (Figure 1a,c vs. Figure 1b,d). Additionally, we compared the proportion of the two variation components explained by rainfall with the unexplained residuals to understand how forage nutrient content is affected by rainfall (Figure 1a,b vs. Figure 1c,d). The same method was also applied to quantify the roles of species turnover and intraspecific variation and the effect of rainfall in the total across‐site variation in nutrient ratios.

All statistical analyses were conducted using R 4.4.1 (R Core Team, 2024). The R script for decomposing variation into species turnover effect and intraspecific variation was based on publicly available code from Götzenberger et al. (2020).

## RESULTS

### Variation in leaf elemental contents between sites and species

**FIGURE 3:**
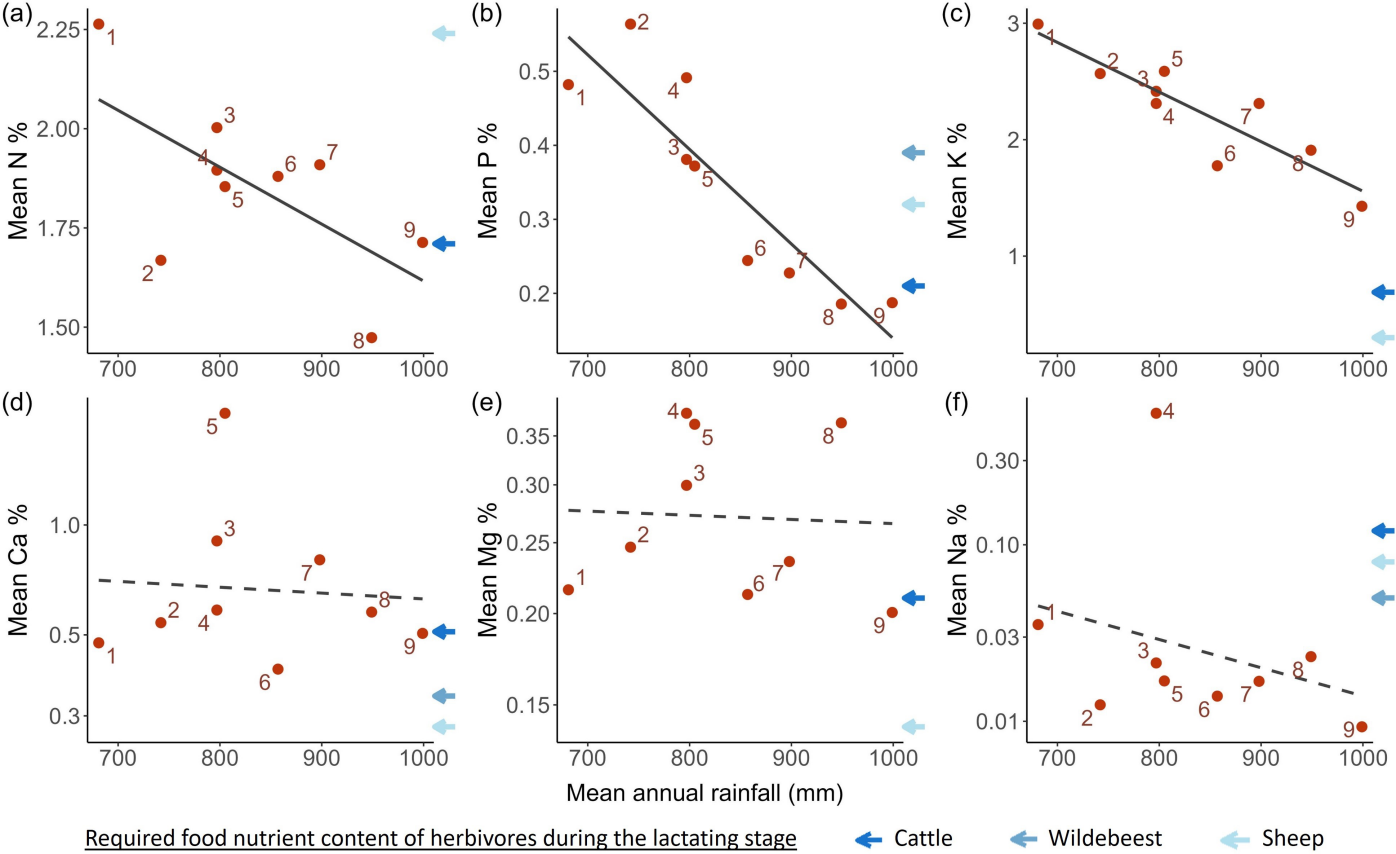
Variation in site‐average leaf elemental content along the rainfall‐soil fertility gradient. The mean annual rainfall was calculated using the CHIRPS dataset (Climate Hazards Group InfraRed Precipitation with Station data) between 2000 and 2021. The weighted mean leaf elemental content decreased along the rainfall‐soil fertility gradient for (a) nitrogen (*R* ^2^ = 0.34, *F*_1,7_ = 5.1, *p* = 0.059), (b) phosphorus (*R* ^2^ = 0.77, *F*_1,7_ = 28.1, *p* = 0.001), and (c) potassium (*R* ^2^ = 0.78, *F*_1,7_ = 28.7, *p* = 0.001). Rainfall was not significantly correlated with leaf content of (d) calcium (*R* ^2^ = −0.14, *F*_1,7_ = 0.04, *p* = 0.85), (e) magnesium (*R* ^2^ = −0.14, *F*_1,7_ = 0.02, *p* = 0.90), or (f) sodium (*R* ^2^ = −0.04, *F*_1,7_ = 0.71, *p* = 0.43). A solid line responds to a *p*‐value <0.1, and a dashed line indicates a *p*‐value ≥0.1. The *y* axes of (d–f) are log_10_ transformed due to the normalization applied in the fitted linear models.

**FIGURE 4:**
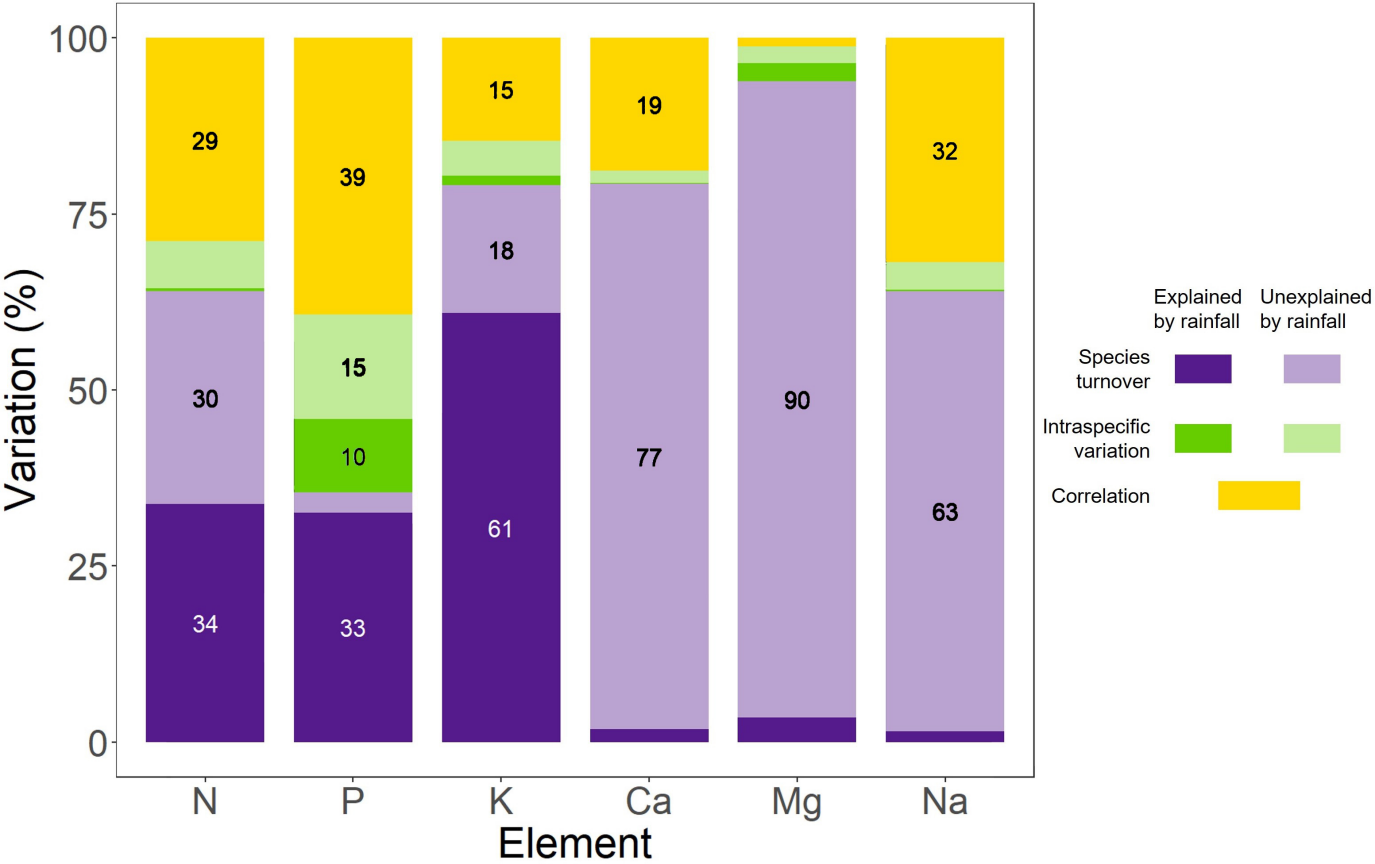
Decomposition of variation in nutrient content across sites. Variation in nutrient content across the sites can be broken down into three components: species turnover effect (purple), intraspecific variation (green), and their covariation (yellow). Both species turnover effect and intraspecific variation can be further divided into the portion related to the rainfall‐soil fertility gradient (dark color) and the portion not related to the gradient (light color). The values (white or black numbers) indicate the percentage of variation explained by each factor, and only values exceeding 10% are presented here.

**FIGURE 5:**
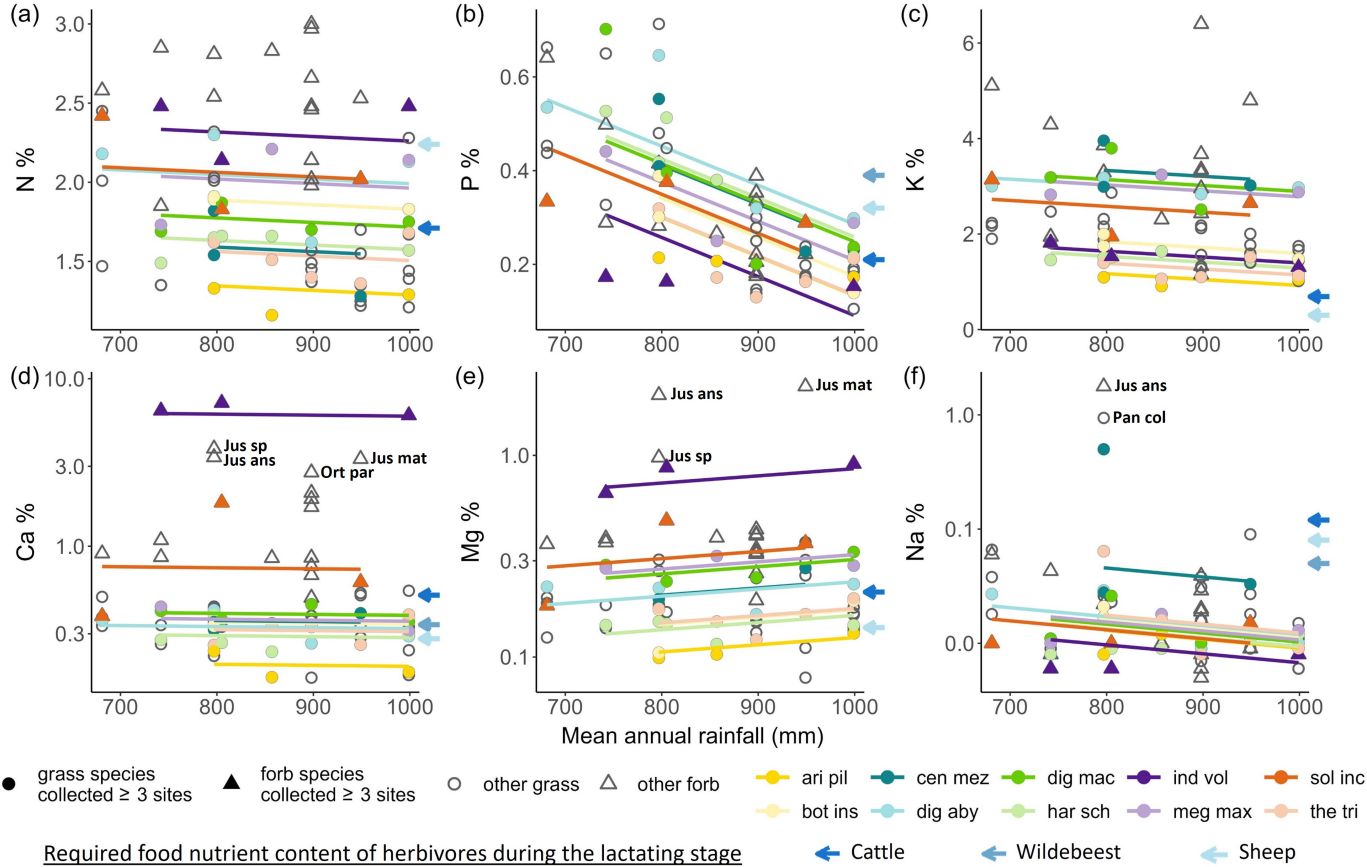
Intraspecific variation of leaf elemental content along the rainfall‐soil fertility gradient. Leaf elemental contents of species collected at more than three sites were fitted in general linear mixed models with species as a random factor for the intercepts to estimate the intraspecific variation along the rainfall‐soil fertility gradient for (a) nitrogen, (b) phosphorus, (c) potassium, (d) calcium, (e) magnesium, and (f) sodium. Leaf elemental content remained relatively constant within species in N (coefficient = −2.9 × 10^−4^, 95% CI = −9.7 × 10^−4^ ~ 3.8 × 10^−4^, *t*_34_ = −0.9, *p* = 0.40), Ca (coefficient = −6.1 × 10^−5^, 95% CI = −4.8 × 10^−4^ ~ 3.6 × 10^−4^, *t*_34_ = −0.3, *p* = 0.77), and Na (coefficient = −7.8 × 10^−4^, 95% CI = −1.8 × 10^−3^ ~ 2.2 × 10^−4^, *t*_34_ = −1.6, *p* = 0.13), decreased with increasing rainfall in P (coefficient = −8.3 × 10^−4^, 95% CI = −1.2 × 10^−3^ ~ −5.1 × 10^−4^, *t*_34_ = −5.1, *p* < 0.001) and K (coefficient = −1.2 × 10^−3^, 95% CI = −2.3 × 10^−3^ ~ −1.4 × 10^−4^, *t*_34_ = −2.2, *p* = 0.03) and increased with rainfall in Mg (coefficient = 3.6 × 10^−4^, 95% CI = 4.6 × 10^−5^ ~ 6.6 × 10^−4^, *t*_34_ = 2.3, *p* = 0.03). The *y* axes of (d–f) are log10 transformed. Uncommon species with notably high Ca, Mg, or Na content are labeled using their abbreviated names: Jus ans, *Justicia anselliana*; Jus mat, *Justicia matammensis*; Jus sp., *Justicia* species; Ort par, *Orthosiphon parvifolius*; Pan col., *Panicum coloratum*. Species name abbreviations in the legend: Ari pil, *Aristida pilgeri*; bot ins, *Bothriochloa insculpta*; cen mez, *Cenchrus mezianus*; dig aby, *Digitaria abyssinica*; dig mac, Digitaria macroblephara; har sch, *Harpachne schimperi*; ind vol, *Indigofera volkensii*; meg max, *Megathyrsus maximus*; sol inc, *Solanum incanum*; the tri, *Themeda triandra*.

Site‐average leaf N, P, and K contents decreased strongly with increasing rainfall and decreasing soil fertility (Figure 3a–c). However, the underlying causes of these declines differed among the elements (Figure 4). For N and K, species turnover was the dominant factor, contributing 9 and 12 times more, respectively, than intraspecific variation to the variation in average nutrient contents across sites (Figure 4). More than half of the species turnover effect was explained by the rainfall gradient (N: 34% out of 64%, K: 61% out of 79%, Figure 4), indicating a vegetation composition change from being dominated by intrinsically high N and K species at low rainfall to low N and K species at high rainfall (Appendix S1: Figure S2). Leaf N and K contents within the same species did not differ significantly across the rainfall‐fertility gradient (Figure 5a,c).

In contrast to N and K, variation in leaf P content across sites was caused by both species turnover and within‐species variation between sites (interspecific: 35%, intraspecific: 25%), and both of them were related to rainfall (Figure 4). With increasing rainfall (decreasing soil fertility), vegetation composition shifted from P‐rich species dominated to P‐poor species dominated (Appendix S1: Figure S2), and meanwhile, individual leaf P content within species strongly decreased with increasing rainfall (Figure 5b). For instance, *Digitaria abyssinica* experienced a 2.2‐fold reduction in P content along the rainfall gradient from about 800 (site 3) to about 1000 (site 9) mm year^−1^ (Figure 5b).

Leaf Ca, Mg, and Na contents varied considerably between sites, but this variation was not clearly related to the rainfall‐fertility gradient (Figure 3d–f). Species turnover between sites accounted for the majority of this variation (Figure 4). Intraspecific contents of these three elements remained mostly constant across the rainfall‐fertility gradient (Figure 5d–f). Additionally, while significant intraspecific variation of Na content was observed within one species, *Cenchrus mezianus*, ranging from 0.50% (site 4) to 0.03% (site 3), no significant directional intraspecific variation with rainfall was detected (Figure 5f).

Ca, Mg, and Na exhibited notably higher leaf contents at specific sites compared to other sites: Ca content peaked at site 5, Mg content was elevated at sites 4, 5, and 8, and Na content was exceptionally high at site 4 (Figure 3d–f). These elevated element contents stemmed from the presence of species notably rich in specific elements, such as Ca‐rich *Indigofera volkensii* at site 5 (Figure 5d), Mg‐rich *Justicia anselliana* and *Justicia* species at sites 4, *Indigofera volkensii* at site 5, and *Justicia matammensis* at site 8 (Figure 5e), and Na‐rich *Justicia anselliana* and *Panicum coloratum* at site 4 (Figure 5f). This underscores that variation across sites in elemental contents primarily arose from changes in species composition.

### Leaf elemental ratios

**FIGURE 6:**
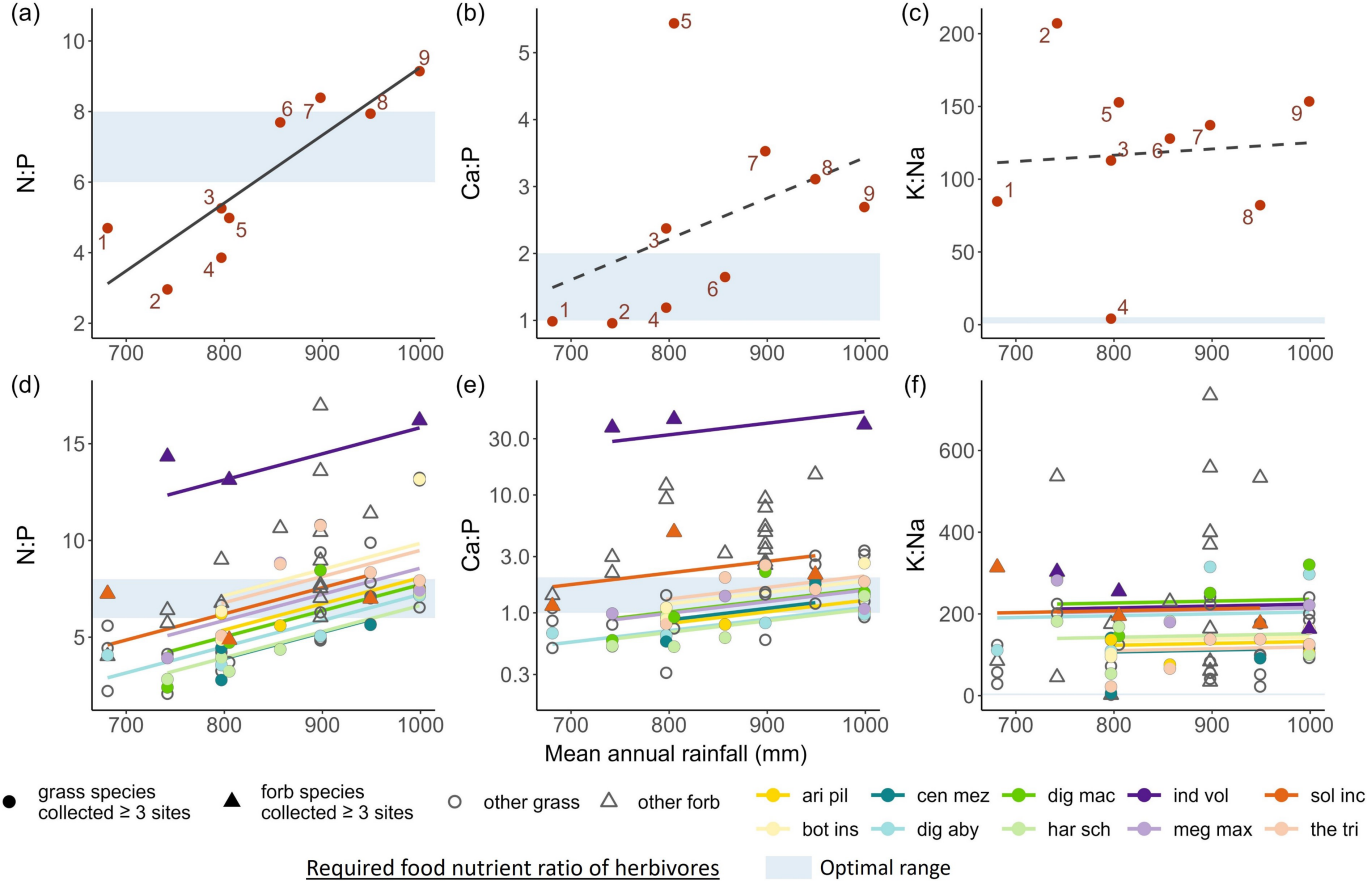
Variation in site leaf elemental ratios and intraspecific elemental ratios across the rainfall‐soil fertility gradient. Site elemental ratios, calculated by the ratio of site weighted mean contents of two elements, increased along the rainfall gradient for (a) N:P ratio (*R* ^2^ = 0.72, *F*_1,7_ = 21.9, *p* = 0.002). Rainfall was not significantly correlated with (b) Ca:P ratio (*R* ^2^ = 0.06, *F*_1,7_ = 1.5, *p* = 0.26) or (c) K:Na ratio (*R* ^2^ = −0.14, *F*_1,7_ = 0.04, *p* = 0.85). Leaf elemental ratio within species increased with rainfall in (d) N:P ratio (coefficient = 0.01, 95% CI = 7.9 × 10^−3^ ~ 1.9 × 10^−2^, *t*_34_ = 4.8, *p* < 0.001) and (e) Ca:P ratio (coefficient = 9.8 × 10^−4^, 95% CI = 4.2 × 10^−4^ ~ 1.5 × 10^−3^, *t*_34_ = 3.5, *p* = 0.001), and remained relatively stable in (f) K:Na ratio (coefficient = 0.05, 95% CI = −2.0 × 10^−1^ ~ 2.8 × 10^−1^, *t*_34_ = 0.4, *p* = 0.71). The *y* axis of (e) is log_10_ transformed. The solid line in (a) responds to a *p*‐value <0.1, and the dashed line in (b) and (c) indicates a *p*‐value ≥0.1. Species name abbreviations in the legend: ari pil, *Aristida pilgeri*; bot ins, *Bothriochloa insculpta*; cen mez, *Cenchrus mezianus*; dig aby, *Digitaria abyssinica*; dig mac, *Digitaria macroblephara*; har sch, *Harpachne schimperi*; ind vol, *Indigofera volkensii*; meg max, *Megathyrsus maximus*; sol inc, *Solanum incanum*; the tri, *Themeda triandra*.

Site leaf N:P ratio strongly increased with increasing rainfall and decreasing soil fertility (Figure 6a), which stemmed from both intraspecific variation (Figure 6d) and species turnover (Appendix S1: Figure S3). Site Ca:P ratio also increased with rainfall but not significantly (Figure 6b). Its variation across sites was primarily explained by species turnover (Appendix S1: Figure S3), despite the intraspecific Ca:P ratio increasing marginally with rainfall (Figure 6e). Site K:Na ratio was independent of rainfall (Figure 6c). The variation in K:Na ratio across sites arose from species turnover (Appendix S1: Figure S3), with intraspecific K:Na ratio remaining relatively stable (Figure 6f).

### Link to herbivore nutritional requirements

Comparing leaf nutrient contents to herbivore nutrient requirements, the results on which elements are limited to herbivores were not significantly different between the lactating and non‐lactating stages. Here, we only present the results for the lactating stage, because the plant samples were collected in the peak wet season when most herbivores in Serengeti are lactating (Hopcraft et al., 2015). The results regarding the non‐lactating stage are provided in supplementary information (Appendix S1: Figures S4 and S5).

The extent to which nutrient content met herbivore demands, as estimated from the literature, varied across the different elements. K content at both site and plant species levels consistently exceeded the estimated nutritional needs of the herbivores (Figures 3c and 5c), suggesting that K is not an important determinant of herbivore food selection in this ecosystem. Leaf N, P, Ca, and Mg contents were higher than the herbivore requirements in some sites and species (Figures 3a,b,d,e and 5a,b,d,e), but lower in others, suggesting that these elements could play a role in dietary selection. Na content in nearly all sites and the majority of plant species was significantly lower than the estimated nutritional demands of the herbivores, with only one site and seven out of 75 plant species samples satisfying their Na requirements (Figures 3f and 5f). This limitation of Na is also illustrated in the K:Na ratio, of which almost all sites and all plant species considerably exceeded the optimal range for herbivores across the rainfall gradient (Figure 6c,f). The N:P ratio at both site and plant species levels was lower than the optimal dietary range of herbivores at low rainfall but exceeded this range at high rainfall (Figure 6a,d), suggesting herbivores should select for high N content diets at low rainfall and for high P content at high rainfall. The Ca:P ratio surpassed the optimal range at high rainfall (Figure 6b,e), suggesting that herbivores should increasingly select for high P content—and thus low Ca:P ratio—along the rainfall‐soil fertility gradient.

## DISCUSSION

We investigated the drivers of variation in forage nutrient quality across the Serengeti National Park to better understand possible partitioning of resources among large herbivores. We found that (1) site‐average N, P, and K contents decline along the rainfall‐soil fertility gradient, (2) the declines of N, P, and K result differently from species turnover and intraspecific variation, (3) site‐average Ca, Mg, and Na contents are independent of the rainfall‐soil fertility gradient, and (4) N, P, Ca, Mg, and Na are all potentially limiting nutrients for herbivores (Table 2). In order to achieve a balanced diet for all potential limiting nutrients, Serengeti herbivores might therefore need to employ a complex strategy of foraging on multiple plant species and multiple sites.

**TABLE 2:** Result summary of drivers of variation in forage nutrient content and degree of limitation to herbivores.

Element or element ratio	Overall trend with rainfall	Intraspecific variability	The main mechanism underlying the variation (hypothesis testing result)	Degree of limitation to herbivores
N	Decreasing	Uniform	Rainfall‐related species turnover (Figure 1a)	Moderately limiting
P	Decreasing	Variable	Rainfall‐related species turnover and intraspecific variation (Figure 1a,b)	Moderately limiting
K	Decreasing	Uniform	Rainfall‐related species turnover (Figure 1a)	Sufficient everywhere
Ca	Unresponsive	Uniform	Rainfall‐unrelated species turnover (Figure 1c)	Moderately limiting
Mg	Unresponsive	Uniform	Rainfall‐unrelated species turnover (Figure 1c)	Moderately limiting
Na	Unresponsive	Mostly uniform^a^	Rainfall‐unrelated species turnover (Figure 1c)	Severely limiting
N:P	Increasing	Variable	Rainfall‐related species turnover and intraspecific variation (Figure 1a,b)	Too low at low rainfall, too high at high rainfall
Ca:P	Marginally increasing	Marginally variable	Rainfall‐unrelated species turnover (Figure 1c)	Suitable at low rainfall, too high at high rainfall
K:Na	Unresponsive	Uniform	Rainfall‐unrelated species turnover (Figure 1c)	Too high everywhere

*Note*: The soil fertility is intercorrelated with rainfall.

^a^ Except for one species (*Cenchrus mezianus*) exhibiting large variation in Na content across sites, the Na content of other plant species remained relatively stable across the rainfall–soil fertility gradient.

The decline in vegetation nutrient contents with increasing rainfall (and decreasing soil fertility)—as we observed for N, P, and K—has been commonly reported from regional to global scales (Breman et al., 1979; Joswig et al., 2022; Maire et al., 2015; Veldhuis, Fakkert, et al., 2016) and can be explained by two nonexclusive processes. First, plants obtain minerals from the soil, and reduced nutrient availability in the soil leads to a corresponding decline in plant nutrient contents (Chapin, 1980). Northern Serengeti soils are old, having undergone mineral weathering, nutrient leaching, and runoff for millions of years, whereas southern Serengeti soils have been intermittently enriched by volcanic ash deposits from the Ngorongoro Volcanic Highlands (Jager, 1982). This variation in soil fertility directly influences nutrient levels, particularly for N, P, and K, which are the most commonly limiting nutrients for plant growth (Chapin, 1980; Chen et al., 2024; McNaughton, 1990). Second, higher water availability promotes plant growth (water is used in photosynthesis) and enhances plant CO_2_ intake (Chaves, 1991; Wright et al., 2001). This results in higher carbon‐to‐nutrient ratios and consequently lower nutrient contents in plants (Seagle & McNaughton, 1993).

While site‐average N, P, and K contents all decrease along the rainfall‐fertility gradient, the mechanisms of their decline differ. Decreases in N and K result from species turnover with minimal intraspecific variation, whereas the decline in P originates from both species turnover and intraspecific variation. These differences can be explained by the various inputs of these elements into a biologically reactive form in the Serengeti soil–plant system. N becomes available to plants via plant‐microbe symbiotic N fixation, as in legumes (e.g., *Vachellia* spp., *Senegalia* spp., and *Indigofera* spp.) or diazotroph‐associated grasses, both converting atmospheric N_2_ into NH_4_^+^ or NO_3_^−^ (Fox‐Dobbs et al., 2010; Högberg, 1986; Ritchie & Raina, 2016). In contrast, reactive K^+^ and HPO_4_^2−^ or PO_4_^3−^ primarily originate from mineral K and P through rock weathering (Schlesinger & Bernhardt, 2020; Vitousek et al., 2010), with P mobilization and uptake facilitated by mycorrhizae (Bolan, 1991). The dominant Serengeti parent materials—granite and gneisses—are composed of orthoclase (K‐feldspar) and biotite, relatively high in K, but are deficient in P (Jager, 1982). The most P‐rich soils are depositions of volcanic ash, concentrated on the Southern Serengeti Plains (Dawson, 1964). These different inputs into biologically reactive forms between the three elements align with the observed patterns of soil nutrient content in the Serengeti: total soil N and exchangeable K^+^ are relatively stable across the Serengeti (Antoninka et al., 2015; Jager, 1982; Propster & Johnson, 2015), whereas total soil P decreases drastically from the south to the north in the transition from ash‐derived to granite‐derived soils (Antoninka et al., 2015; Propster & Johnson, 2015; Ruess & Seagle, 1994). Inherent differences in N, P, and K contents between plant species—as observed in our study—likely reflect different physiological and morphological adaptations related to their water and nutrient economy (Field et al., 1983; Sardans & Peñuelas, 2015; Wright et al., 2001), pointing to the importance of species turnover for all three elements. In addition, the strong soil P gradient—in contrast to N and K—might explain the observed intraspecific variation in P content.

We found the site‐average contents of Ca, Mg, and Na to be not significantly related to the rainfall‐soil fertility gradient. Plant Ca and Mg levels exhibit strong taxonomic differences that remain consistent across various growing environments (Broadley et al., 2004; White et al., 2012), for two main reasons. First, unlike N, P, and K, which are primary limiting nutrients for plants (Chen et al., 2024; Elser et al., 2007; LeBauer & Treseder, 2008; Sardans & Peñuelas, 2015), the availability of Ca and Mg in soil is generally sufficient for plant growth (White & Broadley, 2003). This uncouples plant Ca and Mg contents from soil supply. Second, plant Ca and Mg contents are primarily determined by two physiological features showing a strong phylogenetic signal: the concentration of the polysaccharide pectin in the cell wall and the capacity to compartmentalize excess Ca and/or Mg in vacuoles (Broadley et al., 2004; Hawkesford et al., 2023; White et al., 2012, 2018; White & Broadley, 2003). Root cell wall pectins, carrying negatively charged carboxyl groups, are responsible for the uptake of cations—such as Ca^2+^ and Mg^2+^—from the soil (White & Broadley, 2003). Therefore, plant species with inherently high pectin concentrations—most forb species—can maintain high Ca and Mg contents relatively independent of growing conditions (White et al., 2018). Additionally, the plant species exhibiting exceptionally high Ca content in our study (*Indigofera volkensii*, *Orthosiphon parvifolius*, and all *Justicia* species; Figure 5d) belong to families (Fabaceae, Lamiaceae, and Acanthaceae) known for accumulating large amounts of Ca either as water‐soluble cations or Ca‐oxalate precipitates in vacuoles (White & Broadley, 2003). Overall, taxon‐specific physiological features are the main factors determining plant Ca and Mg contents, which make their levels less dependent on the external conditions.

Despite interspecific variation (i.e., species turnover) being identified as the main driver of Na content variation across sites (Figure 4), unique site characteristics seem to be also important for two reasons. First, *Cenchrus mezianus* showed Na contents 10 times lower at other sites compared to site 4 (Figure 5f). Second, all samples exhibiting high Na content were found exclusively at site 4. This suggests that high Na content is likely the result of a combination of factors, including specific plant species that either require Na for processes like carbon fixation (as seen in the NAD‐ME subgroup of C_4_ plants, Murata & Sekiya, 1992) or have evolved in saline environments (Hamilton et al., 2001; Wasim et al., 2022), as well as unique site characteristics, such as specific lithological or hydrological conditions (Hamilton et al., 2001; Jager, 1982).

We found that N, P, Ca, Mg, and Na are all potentially limiting nutrients for herbivores in the Serengeti. Interspecific differences in herbivore nutritional requirements for these nutrients are likely to drive differential resource use. The limitation of those nutrients creates trade‐offs for herbivores between food quality and foraging efforts, which should cause them to target specific food resources that meet their nutrient needs while being sufficiently abundant (Belovsky, 1997; Ritchie & Olff, 2004). Based on the plant nutrient ratios (Figure 6), Na appears to be much more deficient for herbivores compared to other elements and therefore might play a critical role in dietary resource partitioning if Serengeti herbivores rely on plants for their Na intake (McNaughton, 1988). Because larger herbivores (e.g., buffalo *Syncerus caffer*) require more Na per unit body mass than smaller herbivores (e.g., Grant's gazelle and impala) (Duvall et al., 2023), they might be more selective for Na and consequently more restricted to areas and plant species with high Na levels. Plant species P content is very plastic and shows substantial differences between sites, suggesting that spatial resource partitioning may play a more important role than taxon‐based dietary resource partitioning for P resources. Conversely, N content is found to be stable within plant species, and therefore smaller herbivores (e.g., Thomson's gazelle, Grant's gazelle, and impala), which need diets higher in N (Demment & Van Soest, 1985; Van Soest, 1994), are expected to be more selective for plant species with high N content, while larger herbivores can be more generalist. Similarly, larger herbivores are predicted to select for Ca‐rich species because larger herbivores need more Ca due to their higher skeletal investment (Sterner & Elser, 2002) and variation in plant Ca content is primarily taxon‐based.

McNaughton (1988) found that herbivores concentrated their foraging in areas where vegetation had significantly higher mineral content. This raises a critical question: were those herbivores attracted because of particular plant species present at those sites, or because of other unique site characteristics? Our findings suggest that the answer varies between elements. For P, herbivores need to select the right sites depending on their position along the rainfall–soil fertility gradient. For Na, herbivores need to select the right species at the right sites. For N, Ca, and Mg, herbivores need to select the right species, with N‐rich species more common at low rainfall and high soil fertility. Assuming selection of plant species or site characteristics is strongest for the most limiting elements, our results suggest selection should be strongest for Na‐rich forage, and less relevant for elements in excess, like K. In addition, in this study, we did not find any single plant species rich in all the examined nutrients (Figure 5), and likewise, no individual site contains high levels of all essential nutrients (Figure 3). Therefore, to achieve a balanced diet for all potential limiting nutrients, Serengeti herbivores should employ a spatially complex strategy of foraging on multiple plant species and multiple sites. The large size and spatial heterogeneity of the Serengeti likely therefore facilitate the coexistence of the many different herbivore species found in this ecosystem (Anderson et al., 2008).

## CONCLUSION

We identified key drivers of variation in forage nutrient content along an environmental gradient for six essential elements in a protected savanna landscape. Variations in N, K, Ca, Mg, and Na contents are primarily driven by species turnover across sites, while variation in P content is driven by both species turnover and intraspecific variation across sites. Consequently, interspecific variation in the nutrient requirements of herbivores is expected to drive spatial food partitioning (across sites; for P and Na) and taxon‐based food partitioning (across plant species; for N, Ca, Mg, and Na). We suggest that understanding the drivers of forage quality variation may be key for explaining how herbivores partition resources in landscapes where these nutrients are limited. In the Serengeti, plant Na, N, P, Ca, and Mg contents seem particularly limited for large herbivores, suggesting a key role in explaining herbivores coexistence.

## AUTHOR CONTRIBUTIONS

Yuhong Li, Michiel P. Veldhuis, and Han Olff conceived and designed the study. Yuhong Li, Han Olff, Sanne Piek, Emilian P. Mayemba, and Kelvin R. Shoo collected the data and samples in the field. Yuhong Li and Sanne Piek measured plant elemental contents in the lab. Yuhong Li analyzed the data and led the writing of the manuscript. All authors contributed critically to the drafts and gave final approval for publication.

## CONFLICT OF INTEREST STATEMENT

The authors declare no conflicts of interest.

## Supporting information


Appendix S1:

## Data Availability

Data and code (Li et al., 2025) are available in DataverseNL at https://doi.org/10.34894/0JLGIJ.
