# Supplementary material for: Unraveling the drivers of forage quality variation in the Serengeti
Source: Ecology. 2025 Jul 23;106(7):e70168. doi: 10.1002/ecy.70168 (PMC12287624; doi:10.1002/ecy.70168)
Supplement: Supplementary file 1 — Appendix S1: [file ECY-106-e70168-s001.pdf]

Supporting Information

Ecology

Unraveling the drivers of forage quality variation in the Serengeti

Yuhong Li, Sanne Piek, Emilian P. Mayemba, Kelvin R. Shoo, Michiel P. Veldhuis, Han Olff

Appendix S1

Table S1. The 10 herbaceous plant species that were collected at three or more sites (3-5 sites). The specific sites at which each species was collected are shown. The nutrient contents of those species were used to analyze how leaf nutrient content varied within species between sites across the rainfall-soil fertility gradient. All but *Indigofera volkensii* (Fabaceae) and *Solanum incanum* (Solanaceae) are grasses (Poaceae).

Species name	site 1	site 2	site 3	site 4	site 5	site 6	site 7	site 8	site 9
Aristida pilgeri			x			x			x
Bothriochloa insculpta			x	x					x
Cenchrus mezianus			x	x				x	
Digitaria abyssinica	x		x				x		x
Digitaria macroblephara		x			x		x		x
Harpachne schimperi		x	x		x	x			x
Indigofera volkensii		x			x				x
Megathyrsus maximus		x				x			x
Solanum incanum	x				x			x	
Themeda triandra				x		x	x	x	x

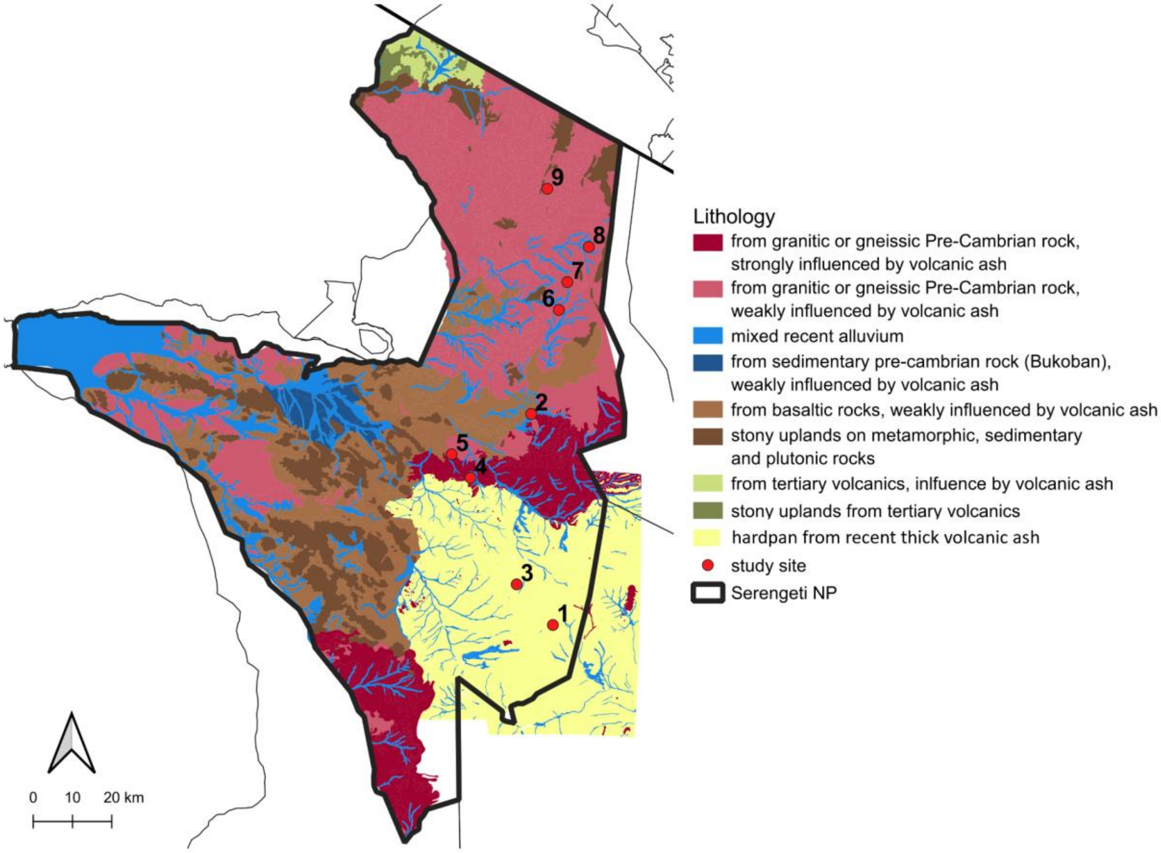

Figure S1. Lithology map of the Serengeti National Park. The study sites are situated on granite or gneiss-derived soil with a decreasing influence of volcanic ash from south to north. The sites were numbered sequentially based on their rainfall levels from lowest to highest (see Figure 1). This map was compiled from information in De Wit (1978) and Jager (1982), combined with our own field observations."

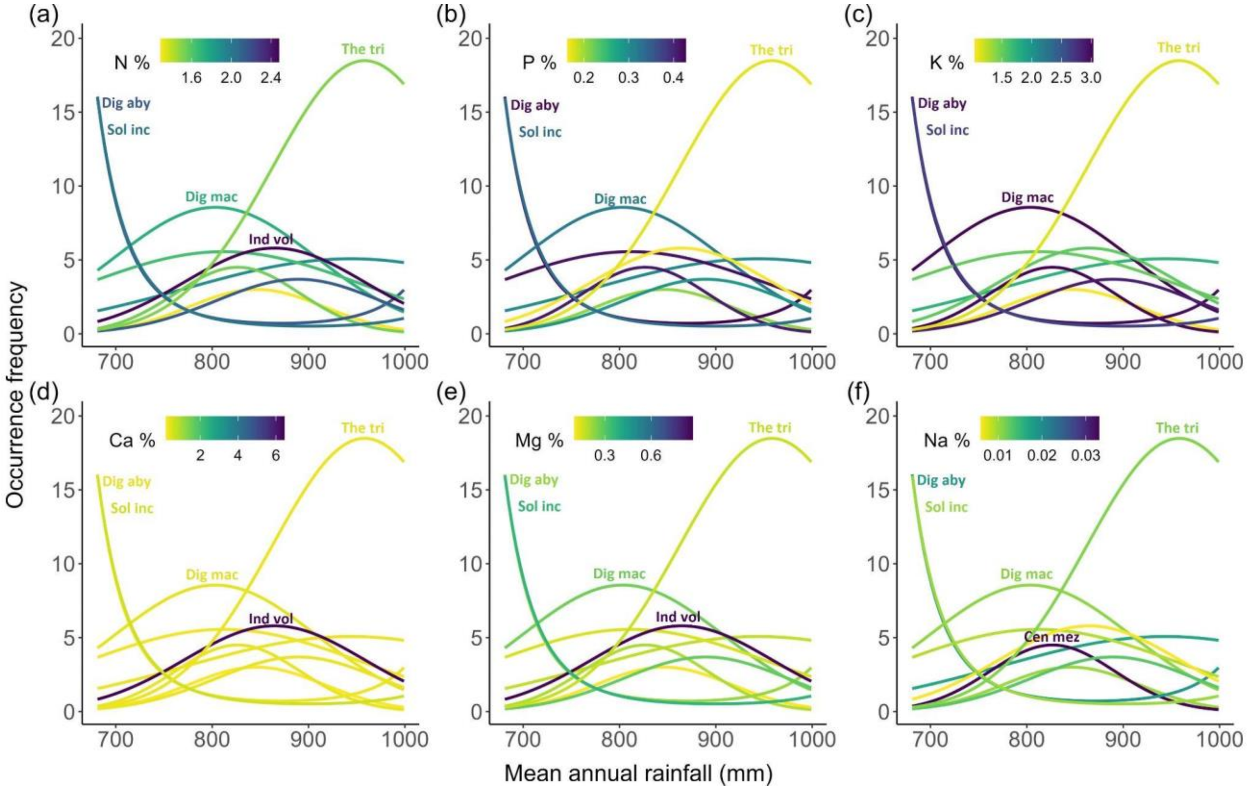

Figure S2. The turnover of the 10 species (species collected ≥ 3 sites) along the rainfall-soil fertility gradient. The occurrence frequency of each species, among the 21 sampling plots along each transect, is shown in fitted quadratic regression (the same in all panels), with coloring representing the species median content of (a) nitrogen, (b) phosphorus, (c) potassium, (d) calcium, (e) magnesium, and (f) sodium. Abbreviated species names are displayed for the most abundant species at different rainfall levels and the species with the highest content within each element. Species name abbreviations in the figure: Cen mez = *Cenchrus mezianus*, Dig aby = Digitaria abyssinica, Dig mac = *Digitaria macroblephara*, Ind vol = *Indigofera volkensii*, Sol inc = *Solanum incanum*, The tri = *Themeda triandra*.

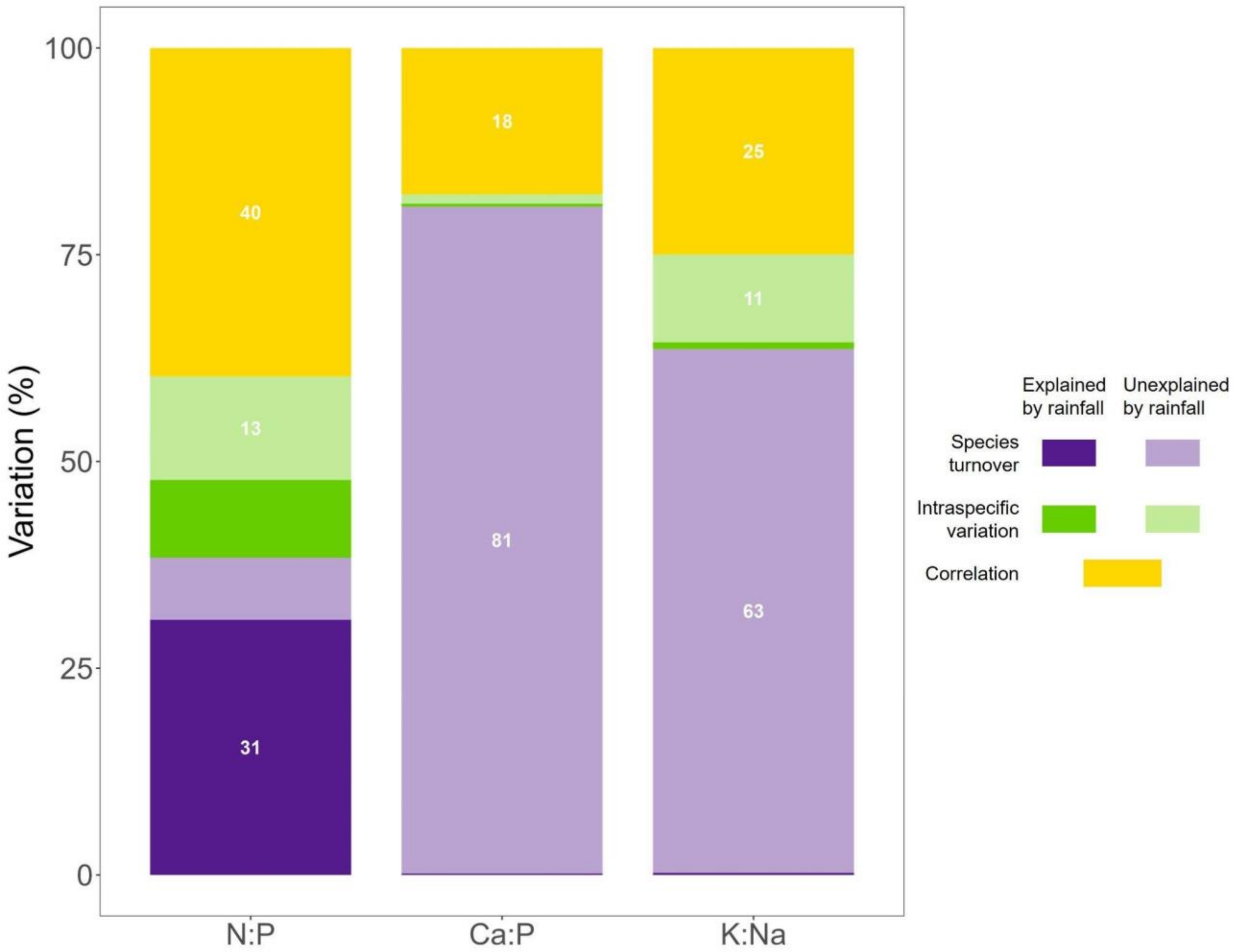

Figure S3. Decomposition of variation in nutrient ratios across sites. Variation in nutrient ratios across the sites can be broken down into three components: species turnover effect (purple), intraspecific variation (green), and their covariation (yellow). Both species turnover effect and intraspecific variation can be further divided into the portion related to the rainfall-soil fertility gradient (dark color) and the portion not related to the gradient (light color). The values (white numbers) indicate the percentage of variation explained by each factor, and only values exceeding 10% are presented here.

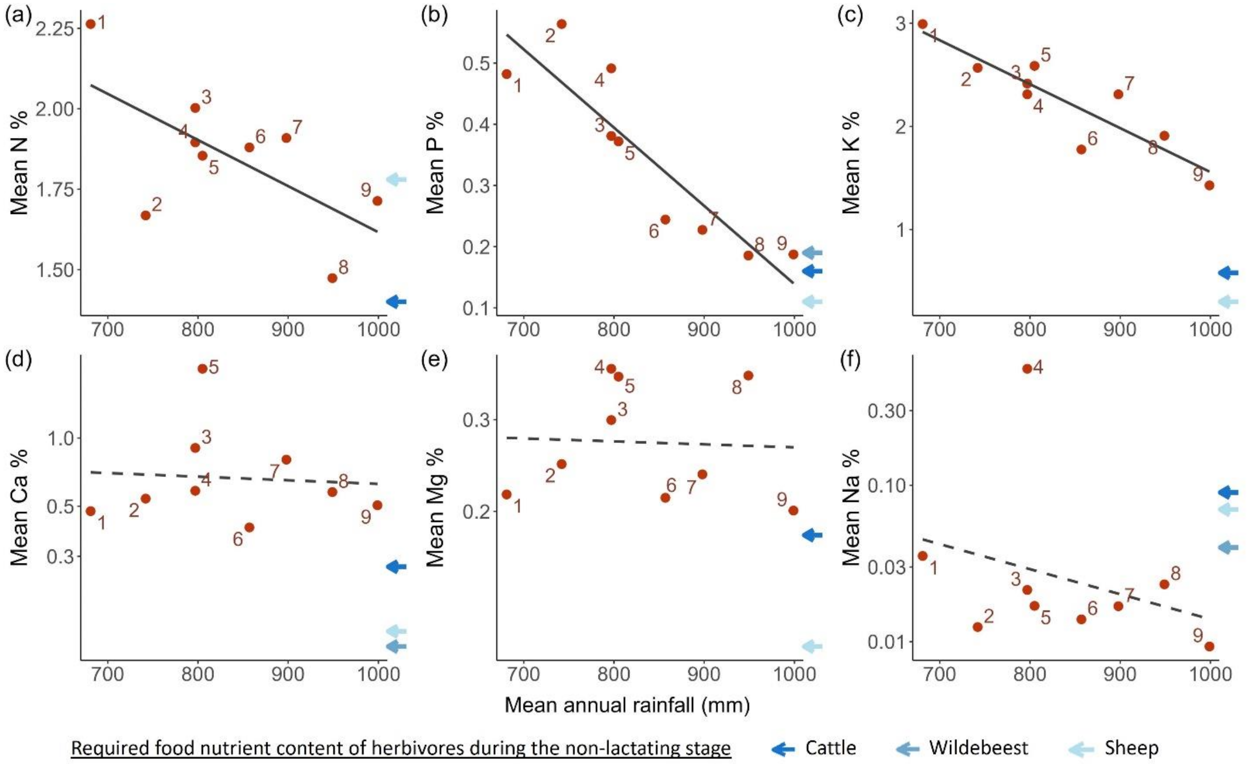

Figure S4. Variation in site-average leaf elemental content along the rainfall-soil fertility gradient, in comparison with herbivore nutrient requirements during the non-lactating stage (National Research Council (U.S.) 1996; Suttle 2010; Murray 1995; Cannas et al. 2004; Kyriazakis and Oldham 1993). Site-average K, Ca, and Mg contents consistently exceeded the nutritional needs of non-lactating herbivores (c–e). Foliar N and P were higher than non-lactating herbivore requirements in some sites, but lower in others (a, b). Na content in nearly all sites failed to meet their nutritional demands (f). The y axes of (d), (e), and (f) are $\log_{10}$ transformed.

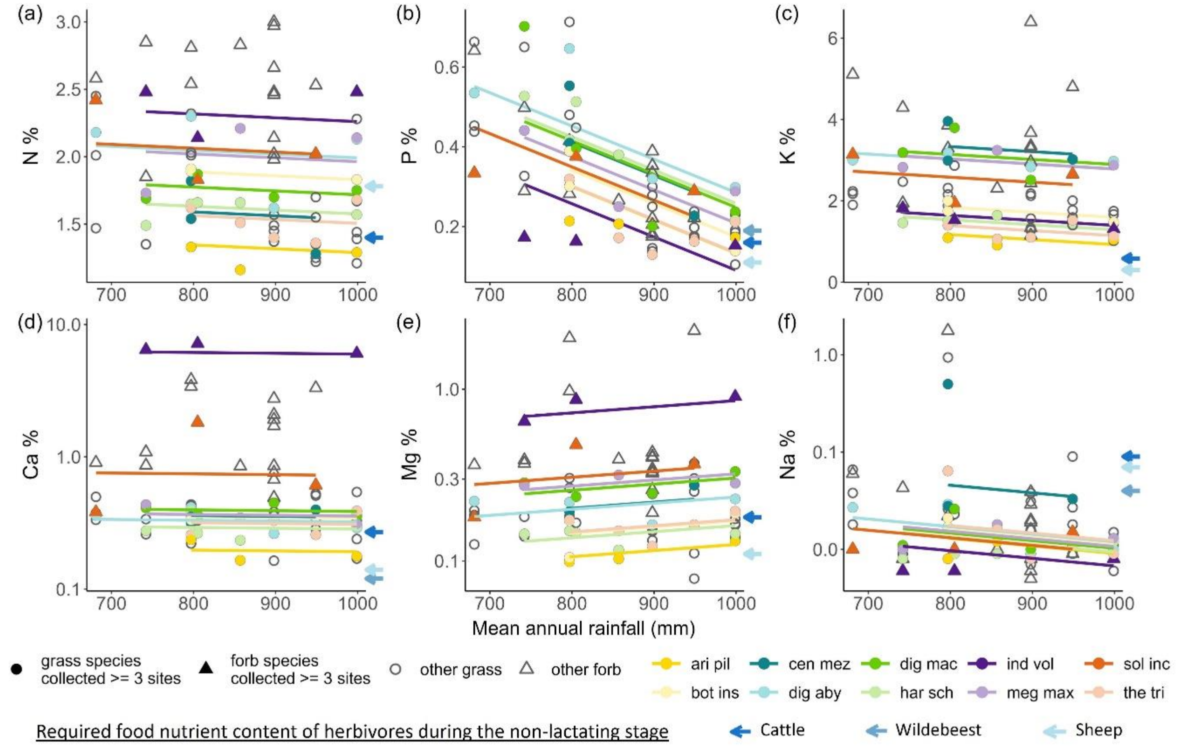

Figure S5. Species-specific intraspecific variation of leaf elemental content along the rainfall-soil fertility gradient, in comparison to herbivore nutrient requirements during their non-lactating stage (National Research Council (U.S.) 1996; Suttle 2010; Murray 1995; Cannas et al. 2004; Kyriazakis and Oldham 1993). K content of all plant species consistently exceeded the nutritional needs of non-lactating herbivores (c). Foliar N, P, Ca, and Mg were higher than herbivore requirements in some species, but lower in others (a, b, d, e). Na content in most of the plant species failed to meet their nutritional demands (f). The y axes of (d), (e), and (f) are $\log_{10}$ transformed. Species name abbreviations in the legend: ari pil = *Aristida pilgeri*, bot ins = *Bothriochloa insculpta*, cen mez = *Cenchrus mezianus*, dig aby = *Digitaria abyssinica*, dig mac = Digitaria macroblephara, har sch = *Harpachne schimperi*, ind vol = *Indigofera volkensii*, meg max = *Megathyrsus maximus*, sol inc = *Solanum incanum*, the tri = *Themeda triandra*.

References

Cannas, A., L. O. Tedeschi, D. G. Fox, A. N. Pell, and P. J. Van Soest. 2004. ‘A Mechanistic Model for Predicting the Nutrient Requirements and Feed Biological Values for Sheep’. *Journal of Animal Science* 82 (1): 149–69. https://doi.org/10.2527/2004.821149x.

De Wit, H. A. 1978. *Soils and Grassland Types of the Serengeti Plain (Tanzania)*. Wageningen: PuDoC.

Jager, T. 1982. *Soils of the Serengeti Woodlands, Tanzania*. Agricultural Research Reports 912. Wageningen: PUDOC.

Kyriazakis, I., and J. D. Oldham. 1993. ‘Diet Selection in Sheep: The Ability of Growing Lambs to Select a Diet That Meets Their Crude Protein (Nitrogen × 6.25) Requirements’. *British Journal of Nutrition* 69 (3): 617–29. https://doi.org/10.1079/BJN19930064.

Murray, M. G. 1995. ‘Specific Nutrient Requirements and Migration of Wildebeest’. In *Serengeti Ii: Dynamics, Management, and Conservation of an Ecosystem.*, edited by A. R. E. Sinclair and P. Arcese, 231–56. Chicago: University of Chicago Press.

National Research Council (U.S.). 1996. *Nutrient Requirements of Beef Cattle*. 7th ed. Washington, D.C.: National Academies Press.

Suttle, N. F. 2010. *The Mineral Nutrition of Livestock*. 4th ed. Wallingford: CABI publ.
